# Prevalence Rates of Personality Disorder and Its Association With Methamphetamine Dependence in Compulsory Treatment Facilities in China

**DOI:** 10.3389/fpsyt.2018.00698

**Published:** 2018-12-12

**Authors:** Chenxi Zhang, Tao Luo, Liang Liu, Huixi Dong, Wei Hao

**Affiliations:** ^1^Hunan Key Laboratory of Psychiatry and Mental Health, Department of Psychiatry & Mental Health Institute of the Second Xiangya Hospital, National Clinical Research Center on Mental Disorders & National Technology Institute on Mental Disorders, Central South University, Changsha, China; ^2^Department of Psychology, JiangXi Mental Health Center, Nanchang, China; ^3^Clinical Psychiatry Department, Wuxi Mental Health Center of Nanjing Medical University, Wuxi, China

**Keywords:** methamphetamine, personality disorder, borderline, antisocial, impulsivity, dependence, addiction

## Abstract

Methamphetamine use is popular and rapidly increasing in China, and the co-occurrence of personality disorders has an impact on treatment outcomes and may increase vulnerability of developing dependence. The aim of the present study was to investigate the prevalence rates of personality disorders in methamphetamine users and further explore the association between personality disorders and methamphetamine use status. Five hundred and seventy-seven male methamphetamine users were recruited. The self-developed questionnaire was used for demographics, and a Structural Clinical Interview for Diagnostic and Statistical Manual of Mental Disorders, Fourth Edition (DSM-IV) (SCID-I/II) was performed covering psychiatric diagnosis. Our study found the prevalence of antisocial personality disorder in male methamphetamine users was 71.4%, followed by borderline (20.2%) and obsessive-compulsive (17.9%) personality disorder. Borderline and antisocial personality disorders were found to be risk factors of methamphetamine dependence (adjusted odds ratio = 2.891, *p* = 0.007 and adjusted odds ratio = 1.680, *p* = 0.042). These findings suggested personality disorders were highly prevalent in male methamphetamine users, and the comorbidity of antisocial and borderline personality disorders are especially associated with methamphetamine dependence.

## Introduction

Novel psychoactive substances (NPS) are synthetic substances that have been developed to produce altered states of consciousness and perceptions. The use of NPS has harmful outcomes on both physical and neuropsychiatric symptoms including respiratory depression, cardiac arrest or multiple organ failure (1). NPS users has higher risk of violence, assaultive, or aggressive behavior requiring restraint when compared with non-NPS users (2). Other symptoms of NPS include sedation and loss of consciousness, to bizarre repeated motions, to erratic running (3). As one of the most commonly known NPS (4), Methamphetamine (MA) has become the second-most prevalent used drug around the world (5). In China, the recreational use of MA is increasing and does not show signs of decreasing (5). In the year 2016, the MA-related seizures reached 29 tons and more than half of the newly reported drug users were MA users in China (6).

The poor treatment outcome and high relapse contributed to the prevalent abuse of MA (7). The comorbidity of personality disorders (PDs) negatively influenced the treatment of dependence (8) and relapse (9) in drug users including MA users (10). PDs are a class of mental disorders characterized by an enduring collection of behavioral patterns often associated with considerable personal, social and occupational disruption (11). Longitudinal studies showed that co-occurrence of PD in drug dependent users had impact on more problems (higher levels of crime, injection-related health problems), more severe drug use (overdose) and other psychiatric problem (major depression) (12, 13). Thus, better knowledge of PDs in MA users may help to improve the detoxification treatment and lower the relapse rate, and prevent the later negative outcomes of the comorbidity.

However, no epidemiologic survey has been reported on the comorbidity of PDs in a homogeneous group of methamphetamine users in the world. Previous studies showed high comorbidity of PD and substance use disorders (SUDs). The overall rates of PD among patients with SUD ranged from 50 to 92% (14). Unfortunately, these data on PDs prevalence in SUD population were not well-applicable to MA users. Personality profiles were reported to link the preferential choice of drugs (15), which indicating different prevalence rates of PDs in different types of drug users. It is also supported by another 1-year longitudinal study, which found that MA users were significantly different from alcohol and marijuana users on PD related factors (16), such as the respect to completion of and readmission to treatment, employment and various forms of criminal justice involvement (17).

The dependence on MA was one of the main reasons for high relapse in MA users (18). As a strong central nervous system (CNS) stimulant, MA is known to have a high dependence liability. MA dependence is a chronic relapsing disorder characterized by compulsive MA use, loss of control over intake, and impairment in social and occupational function (19). PD increased the possibility for developing dependence in drug users (20, 21). However, no studies reported the association between MA dependence and PDs.

Therefore, our study tried to investigate the prevalent rates of PDs in MA users and further explore the association between PD and MA dependence. Our study may provide evidence of the PD prevalence in MA users in compulsory detoxification facilities in China. The association between PD and the dependence of MA may help to offer evidence for the development from MA use to dependence.

## Methods

### Participants and Design

The 577 participants in this study were from two male compulsory detoxification treatment facilities (including Xinkaipu and Bainihu) from July 2013 to November 2013 in Hunan province, China. Compulsory rehabilitation is the primary form of treatment for illegal drug dependence in China (61.6% drug users received compulsory treatment by 2012) (22). The residential MA users were admitted in the treatment facilities after confirmed MA use by urine or hair test. Routine physical exams were performed before the admission. There were 52 MA users (9.0%) excluded because of uncompleted data. In the end, 525 MA users remained in the study. Inclusion criteria of MA users were as follows: age ≥18 years old, admitted compulsively because of MA use; mainly used MA and did not use heroin at least 1 year before admission, and able to give consent. Those MA users with current psychotic symptoms were interviewed after these symptoms disappeared. The exclusion criteria were as follows: having severe somatic disorders and any history of severe brain diseases, such as brain trauma, epilepsy, or encephalitis. To explore the association, the MA users were then divided based on the results of Diagnostic and Statistical Manual of Mental Disorders, Fourth Edition (DSM-IV) diagnosis into two groups: MA-dependent and non-dependent users. All subjects expressed a wish to participate in the study in order to receive medical examinations from the psychiatrists conducting the investigation, out of concern for their own health. They were free to abstain from participation in the study or to withdraw from it without threat of punishment. All the participants freely gave informed consent prior to their inclusion in the study. The research protocol and informed consent were approved by the Human Ethics Committee of the Second Xiangya Hospital of Central South University.

### Procedure

Sociodemographic data were collected initially. Then, face-to-face interviews were conducted by three trained psychiatrists. Structured Clinical Interviews for DSM-IV Axis I disorders and personality disorders (SCID-I/II), Chinese version (23) were used for the diagnoses; there was a good inter-rater reliability of (kappa = 1.0) among the three psychiatrists. Approximately 1.5–2 h were utilized for the whole interview.

### Statistical Analyses

All the statistical analyses were entered into SPSS (version 22.0) for Windows. Means and frequencies were computed. Group comparisons were performed between MA-dependent and non-dependent users using Independent *t*-test, Chi-square test and Fisher exact test as appropriate. The association between each PD and MA dependence was evaluated where there were significant group differences between PDs. Relationships between factors and one binary outcome variables (MA-dependent and non-dependent users) were tested with multiple logistic regression models, controlling for demographics (age), Axis I disorders (psychotic disorder, dependence of hypnotics/heroin/ketamine) and MA use pattern (onset age of MA use, duration of MA use and routes of MA administration). Each multiple logistic regression model produced adjusted odds ratios and 95% confidence intervals for the PD. The level of significance was set at 0.05 in all analyses.

## Results

Five hundred and twenty-five male MA users were analyzed in the study. Table 1 showed the sociodemographic data and lifetime diagnoses of Axis I disorders of the study sample and the differences between the two subgroups. The MA users suffered from affective disorder (26.1%), followed by psychotic disorder (20.2%), and anxiety disorder (7.2%). Alcohol dependence was diagnosed in 17.5% MA users. MA users also used other drugs and were diagnosed with a lifetime dependence, such as heroin (34.1%), hypnotics (9.1%), ketamine (8.2%), cannabis (1.5%), and cocaine (0.4%). The MA use pattern was shown in Table 2. The average age of the first MA use was 23.3 years old. The mean duration of MA use was 32.6 months among all MA users. Most of the MA users (74.5%) used MA by smoking. The prevalence rate of PD among male MA users was highest for antisocial (71.4%), followed by borderline (20.2%), obsessive-compulsive (17.9%), avoidant (16.2%), paranoid (14.3%), negativistic (14.1%), narcissistic (6.3%), histrionic (5.5%), schizoid (5.1%), dependent (1.5%), and schizotypal (0.6%) PDs (Table 3).

**Table 1:** Demographic and clinical data of the study sample.

	**All MA users (*n* = 525)**	**MA-dependent users (*n* = 388)**	**MA non-dependent users (*n* = 137)**	***p*-value**
**DEMOGRAPHICS**				
Age	33.3 ± 7.2	31.8 ± 7.2	33.7 ± 7.0	0.006^a^
Married (0 = no, 1 = yes) (*n*, %)	210 (40.2)	159 (41.4)	51 (37.5)	0.463^b^
Educational level				0.675^b^
Illiteracy/primary school (*n*, %)	112 (21.4)	82 (21.2)	30 (21.9)	
Junior middle school (*n*, %)	302 (57.6)	227 (58.7)	75 (54.7)	
Senior middle school and above (*n*, %)	110 (21.0)	78 (20.2)	32 (23.4)	
**AXIS I DISORDER**				
Affective disorder (*n*, %)	137 (26.1)	103 (26.5)	34 (24.8)	0.692^b^
Major depressive disorder (MDD)	106 (20.2)	77 (19.8)	29 (21.2)	0.740^b^
Current MDD	33 (6.3)	22 (5.7)	11 (8.0)	0.328^b^
Past MDD	78 (14.9)	58 (14.9)	20 (14.6)	0.921^b^
Mania	45 (8.6)	38 (9.8)	7 (5.1)	0.092^b^
Current mania	0	0	0	–
Past mania	45 (8.6)	38 (9.8)	7 (5.1)	0.092^b^
Dysthymia disorder	11 (2.1)	7 (1.8)	4 (2.9)	0.489^c^
Bipolar disorder	43 (8.2)	36 (9.3)	7 (5.1)	0.126^b^
Anxiety disorder (*n*, %)	38 (7.2)	32 (8.2)	6 (4.4)	0.133^b^
Panic disorder	3 (0.6)	2 (0.5)	1 (0.7)	1.000^c^
Agoraphobia without panic	1 (0.2)	1 (0.3)	0	1.000^c^
Social phobia	0	0	0	–
Specific phobia	2 (0.4)	2 (0.5)	0	1.000^c^
Obsessive compulsive disorder	13 (2.5)	12 (3.1)	1 (0.7)	0.200^c^
Post-traumatic stress disorder	7 (1.3)	4 (1.0)	3 (2.2)	0.384^c^
Generalized anxiety disorder	4 (0.8)	4 (1.0)	0	0.577^c^
Substance induced anxiety disorder	10 (1.9)	9 (2.3)	1 (0.7)	0.466^c^
Psychotic disorder (*n*, %)	106 (20.2)	88 (22.7)	18 (13.1)	0.017^b^
Schizophrenia	21 (4.0)	18 (4.6)	3 (2.2)	0.208^b^
Schizophreniform disorder	4 (0.8)	2 (0.5)	2 (1.5)	0.280^c^
Schizoaffective disorder	0	0	0	–
Delusional disorder	6 (1.1)	6 (1.5)	0	0.347^c^
Brief psychotic disorder	1 (0.2)	1 (0.3)	0	1.000^c^
Substance induced psychotic disorder	54 (10.3)	43 (11.1)	11 (8.0)	0.312^b^
GMC induced psychotic disorder	1 (0.2)	1 (0.3)	0	1.000^c^
Psychotic disorder not otherwise specified	19 (3.6)	16 (4.1)	3 (2.2)	0.427^c^
Alcohol dependence (*n*, %)	92 (17.5)	61 (15.7)	31 (22.6)	0.068^b^
Hypnotics dependence (*n*, %)	48 (9.1)	44 (11.3)	4 (2.9)	0.003^b^
Cannabis dependence (*n*, %)	8 (1.5)	8 (2.1)	0	0.119^c^
Heroin dependence (*n*, %)	179 (34.1)	146 (37.6)	33 (24.1)	0.004^b^
Cocaine dependence (*n*, %)	2 (0.4)	2 (0.5)	0	1.000^c^
Ketamine dependence (*n*, %)	43 (8.2)	40 (10.3)	3 (2.2)	0.003^b^

*MA, Methamphetamine*.

a *Independent t-test*.

b *Chi-square test*.

c *Fisher exact test*.

**Table 2:** Methamphetamine (MA) use pattern of the study sample and group comparison between MA-dependent and non-dependent users.

**MA use pattern**	**All MA users (*n* = 525)**	**MA-dependent users (*n* = 388)**	**MA non-dependent users (*n* = 137)**	***p*-value**
Age of first use	23.3 ± 6.4	22.8 ± 6.2	24.9 ± 6.6	0.001^a^
Duration of regular use (month)	32.6 ± 24.8	35.4 ± 24.9	24.6 ± 22.6	< 0.001^a^
Routes of administration				< 0.001^b^
Snorting (*n*, %)	25 (4.8)	3 (0.8)	22 (16.1)	
Smoking (*n*, %)	391 (74.5)	310 (79.9)	81 (59.1)	
Swallowing (*n*, %)	109 (20.8)	75 (19.3)	34 (24.8)	

*MA, Methamphetamine*.

a *Independent t-test*.

b *Chi-square test*.

**Table 3:** Prevalence rates of personality disorders (PDs) of the study sample and group comparison between methamphetamine (MA)-dependent and non-dependent users.

**PD**	**All MA users (*n* = 525)**	**MA-dependent users (*n* = 388)**	**MA non-dependent users (*n* = 137)**	***p*-value**
Schizotypal (*n*, %)	3 (0.6)	3 (0.8)	0	0.571^a^
Dependent (*n*, %)	8 (1.5)	6 (1.5)	2 (1.5)	1.000^a^
Schizoid (*n*, %)	27 (5.1)	13 (3.4)	14 (10.2)	0.002^b^
Histrionic (*n*, %)	29 (5.5)	25 (6.4)	4 (2.9)	0.121^b^
Narcissistic (*n*, %)	33 (6.3)	28 (7.2)	5 (3.6)	0.139^b^
Negativistic (*n*, %)	74 (14.1)	58 (14.9)	16 (11.7)	0.344^b^
Paranoid (*n*, %)	75 (14.3)	61 (15.7)	14 (10.2)	0.114^b^
Avoidant (*n*, %)	85 (16.2)	67 (17.3)	18 (13.3)	0.259^b^
Obsessive-compulsive (*n*, %)	94 (17.9)	77 (19.8)	17 (12.4)	0.051^b^
Borderline (*n*, %)	106 (20.2)	92 (23.7)	14 (10.2)	0.001^b^
Antisocial (*n*, %)	375 (71.4)	293 (75.5)	82 (59.9)	< 0.001^b^

*MA, Methamphetamine; PD, Personality Disorder*.

a *Fisher exact test*.

b *i-square test*.

**Figure 1:**
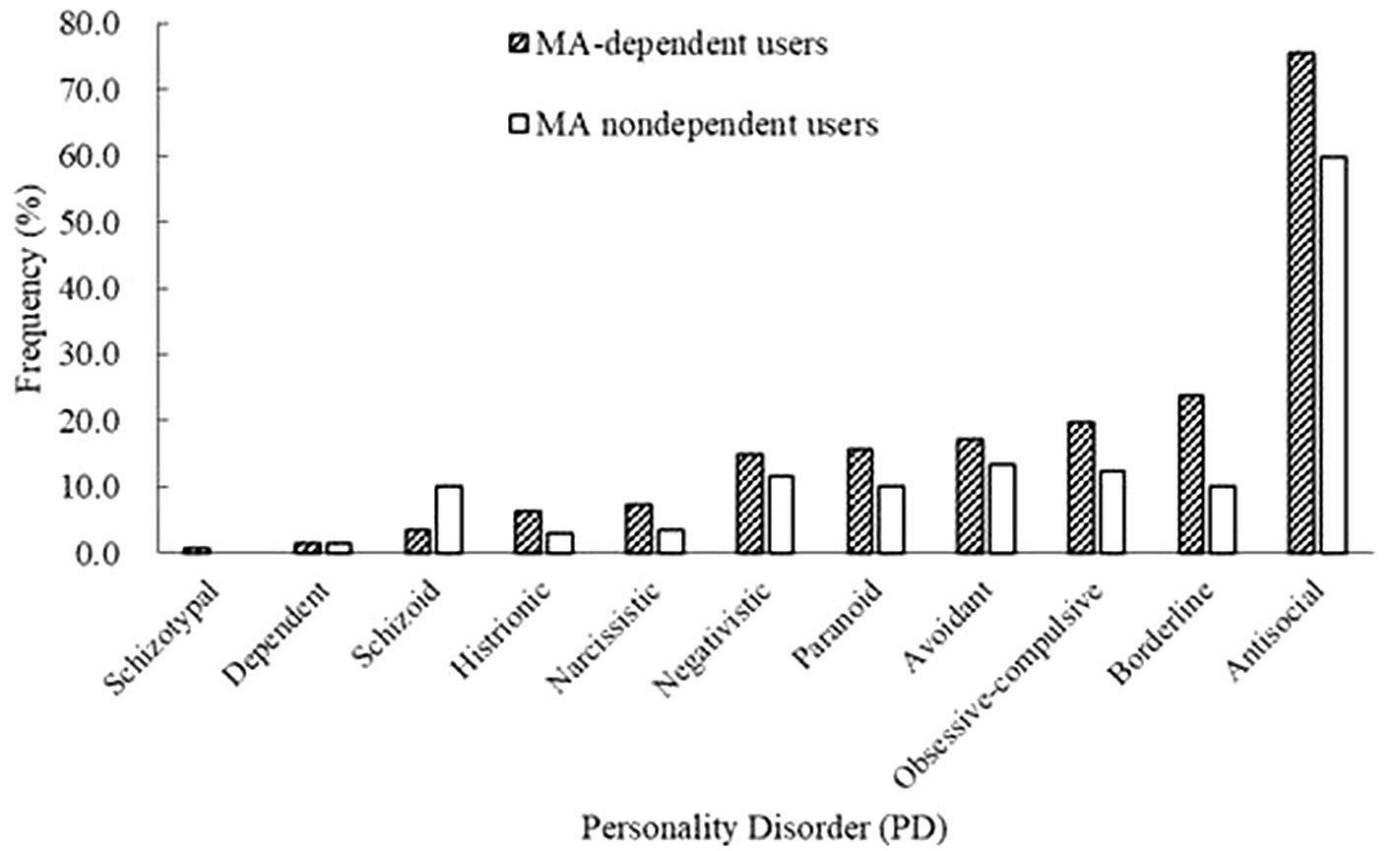
Prevalence rates of personality disorders (PDs) between methamphetamine (MA)-dependent users and non-dependent users.

Among all the MA users, 73.9% of MA users were diagnosed with MA dependence. The MA-dependent users were older than MA non-dependent users (mean age = 33.7 vs. 31.8, *p* = 0.006). There were no significant differences in marriage and education levels between the two groups. Significantly more MA-dependent users were diagnosed with a past psychotic disorder (22.7 vs. 13.1%, *p* = 0.017) and dependences of hypnotics (11.3 vs. 2.9%, *p* = 0.003), heroin (37.6 vs. 24.1%, *p* = 0.004), and ketamine (10.3 vs. 2.2%, *p* = 0.003). No other significant differences were found for the diagnoses of Axis I disorders. A younger onset age of MA use was observed in MA-dependent users compared with those in the MA non-dependent user group (mean age = 22.8 vs. 24.9, *p* = 0.001). MA-dependent users had markedly longer duration of MA use than MA non-dependent users (35.4 vs. 24.6 months, *p* < 0.001). In addition, there were significant differences of routes of administration (*p* < 0.001) between the two groups (MA-dependent and non-dependent users). The different diagnoses rates of PDs between MA-dependent users and MA non-dependent users were presented in Figure 1. There were significant group differences of three PDs, which included schizoid (*p* = 0.002), borderline (*p* = 0.001) and antisocial (*p* < 0.001) PD. The results of the multiple logistic regression model analyses were shown in Table 4, testing the associations of the three PDs with MA dependence after controlled for age, diagnoses of a past psychotic disorder, dependences of hypnotics/heroin/ketamine, age of first MA use, duration of MA use (months) and routes of MA administration. Borderline and antisocial PDs were both risk factors for MA dependence.

**Table 4:** Multivariate logistic regression models to estimate the association between each personality disorder (PD) and methamphetamine (MA) dependence.

**PD**	**AOR^a^**	**95% CI**	***p*-value**
Schizoid	0.483	0.177–1.316	0.155
Borderline	2.891	1.334–6.262	0.007
Antisocial	1.680	1.019–2.771	0.042

*PD, Personality Disorder; AOR, Adjusted Odds Ratio; CI, Confidence Intervals*.

a *Controlled for age, past psychotic disorder, dependences of hypnotics/heroin/ketamine, age of first MA use, duration of MA use (month) and routes of MA administration*.

## Discussion

### Prevalent Rates of Personality Disorders in MA Users

Our study showed much higher prevalence rates of PDs among male MA users than general population (24, 25), schizophrenia patients (26), and female prison inmates (27). The rank of the PDs' prevalence in male MA-dependent users was consistent with a report for male heroin-dependent users in the same setting in China (28). The present study showed relatively higher rates of PDs when compared with Yang's study (28), such as antisocial PD (75.5 vs. 54.2%), borderline PD (23.7 vs. 22.7%), avoidant PD (17.3 vs. 13.9%), paranoid PD (15.7 vs. 8.5%), and negativistic PD (14.9 vs. 11.9%). These differences of PD rates could be mainly due to the different populations (MA vs. heroin). In a study of depressed patient with and without borderline PD, genetic association was found between dopamine transporter (DAT1) and borderline PD (29). Another study found serotonin receptor gene (5-HTT) was associated with BPD gene among BPD patients and healthy controls (30). Antisocial PD was also found associations with dopamine and serotonin transporter genes in alcoholics (31). Thus, borderline or antisocial PD was related to dopamine and serotonin transporters. MA had adverse effects on dopaminergic and serotonin neurons (32) related to PD while heroin affected the opioid receptor (33). Second, the personality trait scores related to the PDs were higher in CNS simulant users (including MA) than those in opioid users (34), which suggested more personality problems for MA users. Third, if use of drug induced PD chronically, then the diagnosis of PD may make a difference at different stages during the clinical course of drug use. Therefore, different clinical courses may also influence the differentiation of PD rates between MA and heroin users. Fourth, the presence of Axis I disorders can influence the prevalence rates of PD including depression (35) and anxiety disorder (36), which were different between our study and Yang's study (28).

### Association Between Personality Disorder and Methamphetamine Dependence

The high prevalence rates of PDs suggested a relationship with MA use. After adjusted with confounding factors, borderline and antisocial PDs were found to be associated risk factors for MA dependence, which were consistently found in patients with substance dependence (37), heroin use (38), and alcohol dependence (39). There were several reasons for the associations between borderline/antisocial PD and MA dependence. First, as a core feature of borderline/antisocial PD, the emotional dysregulation induced more severity of MA use (21). In the present study, MA-dependent users were younger with younger age of first MA use, and they had markedly longer duration of MA use, logically suggested heavier use of MA than MA-non-dependent users. With the repeated severe use of MA, the MA users with borderline/antisocial PD gradually developed to MA dependence. Second, prefrontal cortex (PFC) may play a role in the associations between borderline, antisocial PD and MA dependence. Previous studies showed reduction of gray matter volume in PFC among individuals with borderline PD (40) and antisocial PD (41) when compared with healthy subjects. Prefrontal dysfunction was also found in MA dependent subjects (42). And another study showed that MA-dependent smokers had smaller gray matter volume in the PFC when compared with control non-smokers (43). Thus, PD and the dependence of MA came together to damage the brain region of PFC. In turn, PFC dysfunction induced dysregulation of limbic reward regions (related to addiction) and impairment of higher-order executive function including self-control, salience attribution and awareness (related to PD) (44). MA users with executive dysfunction (45) would also increase the chance to have more severe drug use and became a dependent user easily.

### Possible Role of Personality Disorder During the Development of Methamphetamine Dependence

As a cross-sectional study, the present study cannot clarify the causality between the PDs and MA dependence. There has been reported in a longitudinal study, that the antisocial PD was associated with the later drug dependence at 5 years follow-up (46). In our study, MA-dependent users showed longer duration of MA use than those without dependence, indicating the clinical course of development from MA use to dependence. Therefore, we tried to interpret the role of borderline/antisocial PD during the clinical course of MA use. The first possible reason was borderline/antisocial PD accelerated the clinical course from MA use to dependence. High compulsivity may mediate the transformation from the use to the dependence of MA. As the common feature of borderline and antisocial PDs (47), high compulsivity was also the core feature of dependence (48). The possible pathway was that patients with borderline or antisocial PD has high impulsivity which increased the risk of MA use. Then the chronic use of MA would in turn have impact on borderline or antisocial PD and caused higher impulsivity. The long-term interaction effect of MA use and PD finally induced MA dependence. High impulsivity was found in patients with borderline (49) and antisocial PD (50). Those borderline or antisocial PD patients were susceptible population of MA use (51, 52). MA had neurotoxic effects on human serotonin neurons (53), so did for borderline PD (30) and antisocial PD (31). With chronic use of MA, the MA users with borderline or antisocial PD suffered serotonin deficiencies and had higher impulsivity (54) by genetic vulnerability (55). The dependence of MA finally occurred following the co-occurrence of borderline or antisocial PD in chronic MA users. The other possibility was the MA dependence and borderline/antisocial PD appeared simultaneously with common pathway. In a study of marijuana-dependent users, the comorbidity of antisocial PD and MA dependence was found to increase each other by genetic effect (56). The genetic susceptibility was found between MA dependence and PD mediated by the neuronal cell adhesion molecule (NrCAM) gene variants (57). In addition, dopamine might play a role in the comorbidity of MA dependence and PD (58). A positron emission tomography (PET) study found dopamine increase in PD with addiction group when compared with PD without medication addiction group on the response to a single dose of levodopa (59), suggesting the interaction between addiction and PD.

Our study suggested specific assessment for personality to reduce the possibility from MA use to dependence. For example, Temperament and Character Inventory (TCI) (60) was used in drug dependent users for personality assessment (61). This tool can help elucidate which addiction-associated personality variables are embedded.

There is a need to develop researchers in the area of MA dependence treatment. First, 12-step could be considered as a rehabilitation strategy. A study found that this strategy could provide low- or no-cost options for MA users and increase the capacity for providing treatment (62). Moreover, research showed that the 12-step recovery could be better used for young drug dependent users (63). Our study showed that the onset age of MA use was about 23 years old and their average age was 33 years old. For pharmacotherapy, lisdexamfetamine (LiMA) was recommended for the potential MA dependence treatment (64). Except for the above, Repetitive transcranial magnetic stimulation (rTMS) was also a potential treatment for the MA dependence. The rTMS can directly target and remodel dysfunctions in brain circuits including reward processing, craving, inhibitory and cognitive control, mood, and learning. A pilot study showed that rTMS could help treat the symptom of anhedonia and craving in cocaine users (65).

We have several limitations in our study. First, this is a cross-sectional study that cannot interpret the causality between MA dependence and PD. Second, only male MA users were recruited. The gender effect cannot be detected. Third, the MA users also co-abused other drugs. The impact of other drug use on MA users may exist, even controlled dependences of other drugs.

In conclusion, the comorbidity of PDs in male MA users was prevalent in compulsory detoxification facilities in China. The screening of PDs would be warranted in MA users during admission of detoxification treatment. Most MA users were dependent users in compulsory detoxification facilities in China. Borderline and antisocial PDs were risk factors for MA dependence. The individualized treatment of MA use was suggested with comorbid PD to achieve better treatment outcome and lower relapse rate. The treatment of borderline and antisocial PDs can help to decrease or even prevent dependence of MA.

## Author Contributions

WH conceived and designed this study. CZ, LL, and HD collected the data. CZ conceived and conducted statistical analyses, with additional advice regarding analyses contributed by TL. CZ drafted the manuscript which was approved by all authors.

### Conflict of Interest Statement

The authors declare that the research was conducted in the absence of any commercial or financial relationships that could be construed as a potential conflict of interest.
